# Developing a digital ecological momentary assessment tool for ‘real time’ evaluation in implementation science: testing through evaluation of a novel digital social prescribing intervention

**DOI:** 10.3389/fpubh.2026.1718302

**Published:** 2026-05-22

**Authors:** Ian Tucker, Marcello Bertotti, Ainul Hanafiah, Shahera Hossain, Paul Watts, Md Atiqur Rahman Ahad

**Affiliations:** 1 Department of Psychology and Human Development, University of East London, London, United Kingdom; 2 Institute of Connected Communities, University of East London, London, United Kingdom; 3 Department of Computer Science and Digital Technologies, University of East London, London, United Kingdom; 4 Department of Allied and Public Health, University of East London, London, United Kingdom

**Keywords:** co-design, digital ecological momentary assessment, digital social describing, mixed methods, social prescribing, wellbeing

## Abstract

**Introduction:**

Social prescribing is increasingly adopted as a strategy to address psychosocial determinants of health, yet evaluating complex community-based interventions remains methodologically challenging. Digital ecological momentary assessment (EMA) offers potential for capturing real-time, multi-modal data in naturalistic settings. This study aimed to develop and assess the feasibility of a novel digital EMA approach integrating wearable-derived physiological data with repeated self-report wellbeing measures within a digital social prescribing (DSP) context. Despite increasing interest in EMA and wearable technologies, their integration within social prescribing evaluations remains underexplored.

**Methods:**

A mixed-methods feasibility study was conducted alongside a four-week online chair-based yoga programme delivered through a social prescribing service in East London, UK. Participants wore smartwatches to collect physiological indicators (stress, sleep, heart rate) and completed twice-daily wellbeing assessments using an adapted Short Warwick-Edinburgh Mental Wellbeing Scale. Quantitative data were analysed using exploratory linear mixed-effects models to examine data behaviour and integration within the intensive longitudinal dataset. Participant workshops explored feasibility and acceptability.

**Results:**

Thirteen participants were recruited, with eleven included in quantitative analyses. The study indicated that integrating wearable and self-report EMA data within routine-style DSP delivery is feasible, although challenges were identified regarding device synchronisation, questionnaire adherence, and missing data. Exploratory modelling illustrated substantial within- and between-person variability in wellbeing trajectories and provided an indication of the feasibility of analysing intensive longitudinal EMA data, with no consistent associations between same-day yoga participation and physiological stress markers. Qualitative findings suggested that participants found the approach acceptable and highlighted factors influencing engagement, including flexibility, motivation, and perceived burden.

**Conclusion:**

Integrated wearable and self-report EMA methodologies can be deployed in DSP contexts, but their implementation is associated with important methodological and technical challenges. Findings highlight the need for validated EMA measures, improved data infrastructure, and careful management of participant burden. While not designed to assess intervention effectiveness, this study provides one of the first applied demonstrations of integrating wearable-derived physiological data with EMA self-report measures in a DSP context, offering a methodological foundation for real-time evaluation of complex community-based health interventions.

## 1 Introduction

Social prescribing has expanded rapidly within primary care systems in the UK and internationally as a means of addressing the social determinants of health through referral to community-based activities and services (1–5). Delivered largely through the Voluntary, Community and Social Enterprise Sector (VCSE), social prescribing aims to support individuals experiencing challenges such as loneliness, social isolation, anxiety, and chronic health conditions by facilitating engagement with non-clinical forms of support (6–9). These are recognised drivers of common mental disorders such as anxiety and depression (10, 11). While its policy prominence and uptake have increased substantially (12, 13) the evidence base remains mixed, with ongoing debate regarding both effectiveness and appropriate evaluation methodologies.

A central challenge lies in the complexity of social prescribing interventions (14). These are multi-component, context-dependent, and characterised by heterogeneous participant responses. Conventional evaluation approaches – typically relying on pre-post self-report measures – may not adequately capture the dynamic, individualised, and temporally variable nature of outcomes in real-world community settings. Such approaches are also vulnerable to recall bias and may obscure short-term fluctuations and mechanisms of change (2, 15). There is therefore increasing interest in evaluation methods capable of capturing real-time data within naturalistic contexts.

EMA offers a promising methodological approach in this regard. By collecting repeated self-report data in real-world settings, EMA can reduce recall bias, increase ecological validity, and provide insight into within-person variability over time (16, 17). Despite its growing use in mental health and behavioural research (18), EMA remains underutilised in social prescribing. Barriers include participant burden associated with repeated assessments, limited availability of validated EMA-specific measures, technical challenges in deployment, and the complexity of analysing intensive longitudinal data. These challenges are amplified in community-based implementation contexts, where research control is limited. From an implementation science perspective, such approaches align with calls to capture context-sensitive, real-time variation in intervention delivery and response.

Recent advances in wearable technologies provide further opportunities to extend EMA approaches by enabling continuous, passive collection of physiological data collection alongside self-report measures. Smartwatches can collect indicators related to stress, sleep and heart rate continuously, reducing reliance on participant recall and offering potentially more objective indicators of wellbeing. Integrating wearable-derived physiological data with repeated self-report measures may strengthen evaluation approaches by combining subjective and objective perspectives on health and wellbeing. However, as noted in broader wearable research, such devices can still be affected by adherence issues and intermittent data loss (19, 20, 37) Hence, such integrated approaches remain relatively underused and have not been widely applied in social prescribing contexts. Emerging studies have explored wearable-integrated EMA in areas such as behavioural monitoring and psychiatric populations (21–23), but no studies have examined their feasibility within social prescribing delivery (24, 25). Existing studies have largely examined EMA or wearable data in isolation, with limited integration of multi-modal real-time data within complex community interventions, highlighting a key gap addressed by the present study.

DSP, involving the remote delivery of community-based activities through digital platforms, represents a particularly relevant context for methodological development. DSP has emerged to address barriers to in-person participation, including mobility limitations and accessibility constraints, and may increase reach and scalability (10). However, DSP is in the early stages of implementation, with limited evaluation evidence and ongoing questions regarding its effectiveness and mechanisms of impact (10, 40). DSP is typically delivered via digital platforms (e.g., Zoom, MS Teams) which can offer significant scalability and cost-effectiveness. However, key barriers to implementation exist, which include digital exclusion, variable digital literacy, equipment access and uncertainty about whether digital formats can replicate the peer support and social connection benefits central to social prescribing’s therapeutic mechanism (14). Deploying novel evaluation methodologies within DSP contexts may therefore contribute to both methodological development and the evidence base for digital community interventions.

The current project addressed these gaps by developing and testing a novel digital EMA methodology that integrates wearable-derived physiological data with repeated self-reported wellbeing measures within a DSP setting. The study was conducted alongside a four-week online chair-based yoga programme delivered through a social prescribing service. Yoga is a popular social prescribing activity, which has a significant evidence base regarding its benefits for physical and mental health (26–28). Chair-based yoga delivered online provides an accessible format suitable for individuals with varying physical abilities and health conditions, making it an appropriate real-world context in which to deploy and test digital evaluation approaches.

Importantly, this study is framed as a feasibility investigation. The primary objective was to evaluate recruitment processes, adherence, data completeness, acceptability, and the practical integration of physiological and self-report data streams in real-world delivery conditions. A secondary objective was to explore the behaviour of the resulting intensive longitudinal dataset, including the feasibility of applying mixed-effects modelling and examining temporal patterns of physiological and subjective measures. These included exploring preliminary patterns in wellbeing outcomes and participant experiences associated with the deployment of this methodology to inform methodological development rather than to draw conclusions regarding intervention effects. The methodology involved a co-designed element in which participants contributed to the final study design of a novel digital EMA, which provides significant evidence that digital EMA can become a general evaluation methodology and insight regarding DSP. The co-design element helped to ensure that the final study design was optimized to address our research questions, acceptable to participants and practical, which aligns with current guidance regarding study design in implementation science (29).

By focusing on feasibility and methodological innovation, this study contributes to the development of real-time evaluation approaches for complex community interventions. Specifically, it aims to (1) demonstrate the practical deployment of an integrated EMA system in a DSP context, (2) identify key challenges related to measurement, adherence, and data quality, and (3) provide an initial framework for analysing multi-modal intensive longitudinal data in implementation research. In doing so, the study responds to calls for more ecologically valid and methodologically robust approaches to evaluating social prescribing and similar community-based interventions (30). The study also adds to the limited evidence base regarding DSP activity (4, 14). To our knowledge, this is among the first studies to integrate wearable physiological data with EMA self-report measures within the evaluation of a DSP activity. Conceptually, this study is informed by an implementation science perspective in which intervention engagement, participant behaviour, and measurement processes interact dynamically to shape observed outcomes. The integration of physiological and subjective data is therefore understood not as directly equivalent measures, but as complementary indicators capturing different dimensions of experience.

The study addresses the following research questions:

1. Is it feasible to implement an integrated wearable and EMA-based evaluation approach within a DSP context?
2. What are the key challenges related to adherence, data completeness, and technical integration?
3. How do qualitative participant experiences help contextualise observed quantitative data patterns?

## 2 Methodology

This study employed a mixed-methods feasibility design to evaluate a novel digital EMA methodology integrating wearable physiological data with repeated self-reported wellbeing measures within a real-world DSP context. The DSP activity – a four-week chair-based yoga programme delivered online – provided the implementation setting through which the EMA system was deployed and assessed.

The primary objective was to evaluate the feasibility and acceptability of the digital EMA methodology, including recruitment processes, participant adherence, data completeness, integration of physiological and self-report data streams, and practical challenges associated with real-world deployment. Secondary objectives included exploring preliminary patterns in wellbeing and physiological outcomes and examining participant experiences of both the EMA approach and the DSP activity.

### 2.1 Participants and recruitment

Participants were recruited through a London-based charity that delivers social prescribing activities in East London, UK. A convenience sampling approach was used, with the project advertised to approximately 30 individuals currently engaged with social prescribing activities delivered by the organization. Thirteen participants provided informed consent and enrolled in the study.

Participants were eligible if they were enrolled in or interested in participating in the online chair-based yoga programme and were able to provide informed consent. There were no additional exclusion criteria beyond practical considerations related to participation in digital data collection. Ethical approval was granted by the University of East London (ETH2324-0293) and all participants provided written informed consent prior to data collection.

### 2.2 Digital social prescribing intervention context

The DSP activity consisted of an online chair-based yoga programme delivered twice weekly over four weeks. Sessions were led by an experienced yoga instructor and delivered via an online platform (Zoom), with both live sessions and recorded access available to participants to maximise accessibility and flexibility. The intervention was selected as a suitable real-world context for deploying the EMA methodology due to its accessibility for individuals with diverse physical abilities and health conditions and its relevance within social prescribing services.

The study was not designed to evaluate intervention effectiveness; rather, the DSP programme served as a naturalistic setting in which to test the feasibility of the EMA approach under routine-style delivery conditions.

### 2.3 Digital EMA methodology and data collection

A novel digital EMA methodology was co-designed with participants and involved the integration of three forms of data collection:

1. Physiological data collected continuously via Garmin smartwatches (stress, sleep, heart rate)
2. Repeated self-report wellbeing data collected twice daily using the Short Warwick-Edinburgh Mental Wellbeing Scale (SWEMWBS)
3. Qualitative data collected through participatory co-design and post-intervention feedback workshops.

Participants were provided with Garmin smartwatches to collect physiological data throughout the four-week period. Participants received instruction on device use, including charging and synchronization procedures. Physiological data were processed through a GDPR-compliant digital health solutions platform and returned to the research team for analysis.

Self-report wellbeing data were collected twice daily (morning and evening) using the SWEMWBS delivered via a mobile application (FonLog) (38) for Android users or the Qualtrics survey platform for iOS users. Participants received daily reminders to complete questionnaires. Fixed clock times were not imposed in order to minimize participant burden and reflect the feasibility focus of the study.

The SWEMWBS is a validated 7-item unidimensional measure of mental wellbeing with demonstrated reliability and validity across UK populations (31). The SWEMWBS was adapted for momentary repeated administration despite being validated for retrospective two-week assessment. This decision was informed by the absence of widely validated EMA-specific wellbeing scales suitable for this population and the need to balance brevity with conceptual coverage. The scale was therefore treated as an approximation of momentary wellbeing states rather than a direct extension of its validated use.

Another reason for the use of this scale was our focus on mental wellbeing rather than just mental health and the consolidated evidence of the impact of social prescribing on mental wellbeing using this particular tool (e.g. (32),). We wanted to examine whether this tool would also be useful for this particular ecological study, despite its conventional use over a two-week period rather than twice daily. A single-item self-rated health question was included to capture general health perceptions. Baseline demographic and health information were collected using the mobile application or Qualtrics platform and included age, gender, ethnicity, educational level, employment status, financial difficulty, and long-term health conditions.

### 2.4 Participatory workshops

Two participatory workshops were conducted: a co-design workshop prior to data collection (n = 10 participants, 70 min) and a post-intervention feedback workshop (n = 9 participants, 80 min). Workshops explored participant perspectives on study procedures, accessibility, usability, barriers and facilitators, and perceived impacts of both the EMA methodology and DSP activity.

Workshops were audio-recorded and transcribed verbatim. A semi-structured topic guide was used to ensure consistency across sessions while allowing participants to discuss experiences freely.

### 2.5 Feasibility outcomes

Feasibility was evaluated across multiple domains, included:

- Recruitment and retention rates
- Participant adherence to smartwatch use and EMA completion
- Data completeness and patterns of missing data
- Practical and technical challenges encountered during deployment
- Participant acceptability and experience of the EMA methodology

These outcomes were assessed using quantitative adherence data, monitoring of device usage and questionnaire completion, and qualitative feedback from workshops.

### 2.6 Qualitative analysis

Qualitative data were analysed using reflexive thematic analysis following Braun and Clarke’s framework (34, 35). Two researchers independently coded transcripts, developed initial themes, and discussed the refinement of the thematic structure. Differences in interpretation were resolved through discussion until consensus was reached. Representative participant quotations were selected to illustrate key themes while preserving anonymity.

### 2.7 Quantitative analysis

To explore temporal patterns in physiological and wellbeing outcomes, linear mixed-effects models (LMMs) were fitted separately for stress and SWEMWBS outcomes (39). These models accounted for the hierarchical structure of repeated observations nested within individuals and are robust to unbalanced data and missing observations under the missing-at-random assumption (33, 36). These models assume that missing data are missing at random and that residuals are approximately normally distributed, although formal diagnostic testing was limited due to the small sample size. Analyses were conducted using Mplus (version 8.1) with Full Information Likelihood (FIML) estimation to incorporate all available data.

Given the small sample size and feasibility focus, quantitative analyses were considered exploratory and intended to examine data behaviour rather than to test intervention effectiveness hypotheses. The quantitative analysis also served as a methodological feasibility exercise to assess whether intensive longitudinal EMA datasets generated through this approach could be analysed using LMMs within a small-sample implementation context. Models were specified to explore the inclusion of additional parameters and covariates, including participant characteristics (e.g., age, gender, financial difficulty) and yoga participation as a time-varying variable.

## 3 Results

Results are organised to reflect the feasibility objectives of the study, beginning with participant recruitment and adherence outcomes, followed by quantitative exploratory EMA findings and qualitative insights from the participatory workshops.

### 3.1 Recruitment, retention and feasibility outcomes

Thirteen participants were recruited through a charity in East London that delivers social prescribing following advertisement to approximately 30 individuals engaged with social prescribing services. All recruited participants consented to participate in the study. Two participants were excluded from quantitative analysis due to missing data on all covariates, resulting in a final sample of n = 11 for quantitative modelling. On average, participants did not complete the SWEMWBS measure at approximately 24% of time points (SD = 13.4), with missingness ranging from 0 to 46%. This indicates moderate variability in compliance across participants, with some completing all assessments and others missing nearly half. There was no missing data for the physiological stress outcome, indicating that participants wore the smartwatch consistently enough throughout the study period to generate sufficient daily data for analysis.

Participants had a mean age of 46.8 years (SD 11.0). Most participants were female (10/11, 90.9%), with one male participant (9.1%). Four participants (36.4%) reported financial difficulty at baseline, while seven (63.6%) reported no financial difficulty. Mean baseline stress was 27.7 (SD 12.1), and mean baseline SWEMWBS score was 20.2 (SD 3.2).

Feasibility findings indicated several practical challenges in deploying the digital EMA system in real-world DSP context. These included missed smartwatch synchronization, inconsistent questionnaire completion, and the need for ongoing participant support. However, participants were generally able to engage with the wearable devices and repeated self-report measures, and the study demonstrated the practical possibility of integrating physiological and self-report data streams within routine-style delivery conditions.

### 3.2 Quantitative exploratory EMA findings

All quantitative findings are presented as exploratory indicators of data patterns within this sample and should not be interpreted as evidence of intervention effects. Exploratory linear mixed-effects models were used to examine temporal patterns in wellbeing (SWEMWBS) and physiological stress outcomes across the study period. No *a priori* hypotheses regarding intervention effects were tested, and all quantitative outputs are interpreted as indicators of data behaviour and analytic feasibility only. These analyses were conducted to assess the feasibility of modelling intensive repeated-measure data and to explore data behaviour rather than to draw inferential conclusions. Supplementary Figures 2–5 present observed and model-predicted trajectories for each participant. In these figures, each line represents an individual participant’s scores across their observed study days, with days numbered sequentially from the start of recording.

For wellbeing (SWEMWBS), the initial unadjusted model indicated a small increase in scores over time. However, this pattern was no longer apparent after allowing individual slopes to vary, suggesting between-person variability in trajectories. After adjusting for covariates, younger age was associated with more positive wellbeing trajectories within this sample, whereas gender and financial difficulty were not significantly associated with change over time. Individuals reporting financial difficulty had higher baseline wellbeing scores within this sample. When yoga participation was included as a time-varying covariate, wellbeing scores were lower on days when yoga was completed. This pattern may reflect reverse causality, delayed effects, or temporal misalignment (see Discussion for detailed interpretation). Yoga participation did not significantly moderate the observed rate of change over time.

For physiological stress outcomes, there was no consistent pattern of change observed over time across models. Inclusion of random slopes and covariates did not yield significant predictors of baseline stress or its trajectory. Similarly, same-day yoga participation and its interaction with time were not associated with changes in stress levels. These findings primarily indicate limited detectable signal within this small sample rather than evidence of absence of effects.

Given the feasibility focus and small sample size, these analyses were considered exploratory and intended primarily to examine the behaviour and integration of intensive longitudinal physiological and self-report data rather than to test intervention effectiveness hypotheses.

### 3.3 Qualitative findings from participatory workshops

Qualitative findings provide insight into participant experiences of both the digital EMA methodology and the DSP activity. Three main themes were identified: Access and Flexibility; Motivation and Data Collection Experience; and Personalisation and Intervention Content.

### 3.4 Access and flexibility

Access and flexibility were central to participants’ experiences. The availability of both live and recorded sessions allowed individuals to engage at times that suited work schedules, caregiving responsibilities, and fluctuating health conditions. For participants experiencing anxiety or mobility limitations, participating from home reduced barriers to access. As one participant noted:

Doing it later, the recorded sessions, you could do it in your own time, when it was quiet, so yeah, really, really helpful to have that option.

Participants also valued maintaining a sense of connection to the activity, even when not attending live sessions:

It’s nice to be in the environment itself. But for the days when you can’t be there, I still want to feel involved and feel like I’m getting the benefit from it.

Overall, flexibility was seen as accommodating diverse needs across participants. These perceptions of flexible engagement are consistent with the quantitative finding that yoga participation was not associated with immediate improvements in wellbeing and may reflect participants choosing to engage on days when support was more needed.

### 3.5 Motivation and data collection experience

Participants described the digital EMA tools as influencing motivation and accountability. Some participants reported completing questionnaires and wearing the smartwatch helped them recognise behavioural patterns and maintain engagement:

The questionnaire… was helpful to see the progress actually… I’ve been able to do something consistently.

Another participant highlighted the accountability associated with reporting data:

Having to log back to you guys, give the information back to you guys, made me feel that I would do it.

While many participants preferred digital data collection to paper-based approaches, some found aspects of standardised questionnaire language challenging, particularly terms perceived as vague or emotionally loaded. Technical difficulties with devices and applications were reported, although most participants found these manageable with support. These experiences of motivation and self-monitoring may help contextualise the variability observed in wellbeing trajectories, suggesting that engagement with the EMA process itself may have influenced reported patterns over time.

### 3.6 Personalisation and intervention content

Participants emphasised the importance of personalisation and variation within the DSP activity. Chair-based yoga was viewed as inclusive and accessible, although some participants expressed a desire for greater variation or more physically demanding options, such as standing yoga or other movement-based activities. Flexibility in choosing activities based on energy levels or health status was considered important for sustained engagement.

Beyond the activity itself, participants valued being involved in the co-design process and contributing feedback to the research:

“It made me feel of importance… like what I had to say people wanted to hear.”

The emphasis on tailoring participation to individual needs further supports the interpretation that temporal associations observed in the quantitative data may reflect dynamic and participant-driven engagement patterns rather than uniform responses to the intervention.

Overall, qualitative findings suggested positive perceptions of both the EMA methodology and the DSP activity, with participants reporting perceived benefits for wellbeing, routine formation, and engagement with health behaviours.

## 4 Discussion

This study evaluated the feasibility and acceptability of a novel digital EMA methodology integrating wearable physiological data with repeated self-reported wellbeing measures within a DSP context. Consistent with the study design, the primary focus was methodological feasibility rather than intervention effectiveness. The findings therefore provide insight into the practical implementation, participant experiences, and data characteristics associated with deploying integrated wearable and self-report EMA approaches in real-world community health settings.

### 4.1 Feasibility of the digital EMA methodology

The findings demonstrate that it is practically possible to deploy a multi-modal EMA system combining smartwatch-derived physiological data with repeated self-report measures within routine-style DSP delivery. Participants were generally able to engage with both the wearable devices and survey platforms, and sufficient data were generated to support exploratory intensive longitudinal modelling. These findings suggest that EMA approaches can be implemented in community-based settings outside controlled research environments.

However, feasibility challenges were substantial and have important methodological implications. Inconsistent device synchronization, variable questionnaire completion, and the need for ongoing participant support highlight the dependence of such systems on both technical infrastructure and sustained participant engagement. Missing data were non-trivial and varied across participants, indicating that adherence is a key constraint in real-world EMA deployment. Importantly, these challenges are not incidental but central to assessing feasibility, as they directly influence data quality and interpretability.

Acceptability, as reflected in positive participant feedback, should be considered conceptually distinct from feasibility. While participants reported favourable experiences of both the EMA tools and DSP activity, acceptability does not equate to methodological robustness. Rather, it indicates that the approach is tolerable and potentially engaging, which is a necessary but insufficient condition for reliable data capture and valid inference.

### 4.2 Measurement considerations: adaptation of SWEMWBS for EMA

A key methodological consideration concerns the adaptation of the Short Warwick-Edinburgh Mental Wellbeing Scale (SWEMWBS) for twice-daily EMA use. The scale was originally validated for retrospective assessment over a two-week period, and its use in a high-frequency, real-time context represents a departure from its established psychometric framework. This adaptation was undertaken pragmatically due to the absence of widely validated EMA-specific wellbeing measures suitable for this population and the need to balance brevity with conceptual coverage.

However, this modification introduces uncertainty regarding construct validity and measurement interpretation. Repeated administration at high frequency may alter the meaning of items, shifting responses from reflecting broader mental wellbeing to capturing more immediate affective or situational states. It may also introduce response fatigue or reduce variability due to repeated exposure to identical items. As such, SWEMWBS scores in this study should be interpreted as approximations of momentary subjective wellbeing rather that direct equivalents of the validated scale.

Future research should prioritise the validation of wellbeing measures for EMA contexts or the development of EMA-specific instruments. The present study highlights the practical feasibility of repeated measurement but also underscores the need for stronger measurement foundations in intensive longitudinal research. Future studies may benefit from reduced sampling frequency, adaptive prompting strategies, or passive data augmentation to enhance adherence while maintaining data richness.

### 4.3 Integration of physiological and self-report data

A central innovation of this study was the integration of physiological indicators (stress, sleep, heart rate) with repeated subjective wellbeing measures. The analyses demonstrated that these data streams can be synchronised and modelled within a unified framework, supporting the feasibility of multi-modal data integration in real-world settings.

However, no consistent temporal associations were observed between same-day yoga participation and physiological stress markers, and patterns between physiological and self-reported outcomes were not aligned. Given the small sample size and exploratory nature of the analyses, these findings should not be interpreted as evidence of absence of relationships. Rather, they highlight the complexity of interpreting multi-modal longitudinal data.

Several explanations may account for the observed divergence. First, physiological and subjective measures may capture distinct aspects of experience, with physiological indicators reflecting autonomic processes and self-report measures capturing cognitive appraisal and perceived meaning. Second, temporal misalignment may occur, with physiological responses lagging behind or preceding subjective experience. Third, measurement limitations, including the proprietary nature of wearable-derived stress metrics, may constrain sensitivity to behavioural changes such as yoga participation.

These findings suggest that integration of physiological and subjective data is methodologically feasible but analytically complex. Larger, adequately powered studies will be required to examine temporal relationships more rigorously and to determine whether and how these data streams converge. This finding aligns with previous EMA studies that report weak or inconsistent alignment between physiological and subjective measures in naturalistic settings [e.g. Blaauw et al. (21)].

### 4.4 Interpretations of temporal associations

Exploratory analyses suggested that wellbeing scores were lower on days when yoga was reported as completed. This pattern should be interpreted with caution and not as evidence of a negative effect. Several alternative explanations are plausible. Participants may have been more likely to engage in yoga on days when they were experiencing lower wellbeing (reverse causality), or benefits may manifest after a temporal delay not captured by same-day measurement. Additionally, variability in the timing of questionnaire completion relative to activity participation may have influenced observed associations.

These considerations highlight the importance of careful temporal design and interpretation in EMA studies. Given the twice-daily sampling schedule, the small sample size, and the possibility of reverse causation, these data cannot establish temporal causality or intervention effects. Without controlled timing or lagged analyses, causal inference is not possible, and observed associations should be understood as descriptive temporal co-occurrences. These interpretations are further supported by qualitative findings, which indicate that participants engaged with the activity flexibly in response to their needs, reinforcing the likelihood that observed temporal patterns reflect participant-driven behaviour rather than immediate intervention effects.

### 4.5 Integration of qualitative and quantitative findings

The qualitative findings provide important context for interpreting the quantitative patterns and feasibility outcomes. Rather than representing independent strands of evidence, participant accounts help to explain how behavioural and experiential factors may have shaped observed EMA data.

In particular, participants described engaging with the yoga sessions flexibly in response to their current physical and emotional state. This provides a plausible explanation for the observed pattern of lower wellbeing scores on days when yoga participation was reported, suggesting that engagement may have been reactive to reduced wellbeing rather than preceding improvements.

Participants described increased awareness of routines, motivation, and behavioural engagement associated with the EMA tools, which may help explain observed variability in wellbeing trajectories. These findings suggest that individual differences in engagement with both the DSP activity and the data collection process likely contributed to the heterogeneity observed in the quantitative data.

In addition, reports of questionnaire burden and technical challenges align with patterns of missing data and variable adherence, indicating that data completeness was influenced not only by technical factors but also by participant experience. Taken together, these findings highlight the value of integrating qualitative and quantitative data to understand how implementation processes shape observed outcomes in real-world EMA studies.

Taken together, these findings suggest that the observed quantitative patterns are likely shaped by a combination of behavioural (participant-driven engagement), temporal (timing of effects), and measurement-related (assessment timing and instrument limitations) processes, rather than reflecting direct or immediate effects of the intervention.

### 4.6 Technical challenges and data quality

Technical issues, particularly related to device synchronisation and platform differences, have important implications for data quality. Missing or delayed synchronisation may introduce systematic biases if data loss is associated with participant behaviour (e.g., lower engagement days). As a result, any apparent alignment between physiological and self-report measures should be interpreted cautiously, because timing errors or incomplete synchronisation may have biased completeness and temporal comparability. Similarly, reliance on consumer-grade wearable algorithms, which are often proprietary and not fully transparent, may limit interpretability of physiological measures.

These factors underscore that data generated through digital EMA systems are not neutral but shaped by technical and behavioural processes. Feasibility assessment must therefore include not only whether data can be collected, but how data collection mechanisms may influence the validity and completeness of the dataset.

### 4.7 DSP as a real-world deployment context

The online chair-based yoga programme functioned as a naturalistic setting for testing the EMA methodology. Qualitative findings indicated that digital delivery enhanced accessibility and flexibility, particularly for individuals experiencing mobility limitations or anxiety. Participants valued recorded sessions and flexible engagement patterns, although some reported reduced social connectedness compared to in-person formats. These insights align with broader literature that DSP may increase access while potentially altering social mechanisms of impact.

While participants reported perceived wellbeing benefits, the study was not designed to evaluate intervention effectiveness, and quantitative analyses were exploratory. Instead, the DSP context provided a structured yet ecologically valid environment for testing real-time evaluation methods under conditions approximating routine service delivery.

### 4.8 Implications for implementation science

This study contributes to methodological development in implementation science by investigating the feasibility of deploying integrated EMA systems in community-based interventions such as social prescribing. Traditional evaluation approaches, with particular reference to social prescribing, are typically based on limited time-point assessments (often two time points) and may not capture the dynamic and individualised nature of response in such contexts. Furthermore, traditional evaluation approaches do not typically incorporate physiological data, which may provide important complementary evidence to self-report outcomes. Including digital EMA integrating wearable data suggests a potentially scalable and flexible framework for capturing multi-modal implementation evidence in real-world settings.

However, the findings also highlight key constraints that must be addressed for wider application. These include measurement validity, participant burden, data completeness, and technical infrastructure. The study therefore provides not only a proof-of-concept but also a set of practical considerations for designing and implementing EMA-bases evaluations in real-world contexts.

### 4.9 Limitations

The small sample size limits generalizability and precludes conclusions regarding outcome trajectories. It also restricts the stability of mixed-effects parameter estimates, meaning the quantitative models should be viewed as suggestions of analytic feasibility rather than tests of substantive hypotheses. Missing data, uneven adherence, and technical challenges reflect real-world deployment conditions but constrain statistical inference. The adaptation of SWEMWBS for EMA use introduces additional uncertainty regarding measurement validity. For instance, repeated administration may alter psychometric properties (e.g., response shift, reduced variability, item redundancy), and the construct measured may shift from ‘mental wellbeing’ to short-term affective state. Finally, technical challenges related to device use and data synchronisation may have influenced data completeness and introduced bias. These findings underscore the importance of integrating qualitative insight with intensive longitudinal data to ensure that observed patterns are interpreted within their real-world behavioural and implementation context.

## 5 Conclusion

This study contributes methodologically by demonstrating that integrating wearable-derived physiological data with repeated self-reported wellbeing measures is feasible within DSP contexts, while also identifying key constraints that must be addressed for wider application. The generalisable contribution of this study is the methodological workflow for deploying integrated EMA in routine DSP contexts, rather than the specific intervention results reported here.

The findings highlight that multi-modal EMA systems can generate intensive longitudinal datasets in naturalistic settings, while also identifying key challenges related to measurement, adherence, and data quality. Although the findings are not generalisable in terms of intervention outcomes, the methodological approach is transferable and may inform future studies aiming to evaluate complex interventions in real time.

Further research with larger samples, validated EMA measures, and refined protocols is required to establish the reliability, validity, and broader applicability of this approach for implementation science and social prescribing evaluation.

## Data Availability

The datasets generated and/or analysed during the current study are not publicly available because data sharing was not included in the original ethics approval. Requests to access the datasets should be directed to the corresponding author/s.
