# Supplementary material for: Developing a digital ecological momentary assessment tool for ‘real time’ evaluation in implementation science: testing through evaluation of a novel digital social prescribing intervention
Source: Front Public Health. 2026 May 22;14:1718302. doi: 10.3389/fpubh.2026.1718302 (PMC13236872; doi:10.3389/fpubh.2026.1718302)
Supplement: Supplementary file 1 [file Data_Sheet_1.DOCX]

**Supplementary Materials**


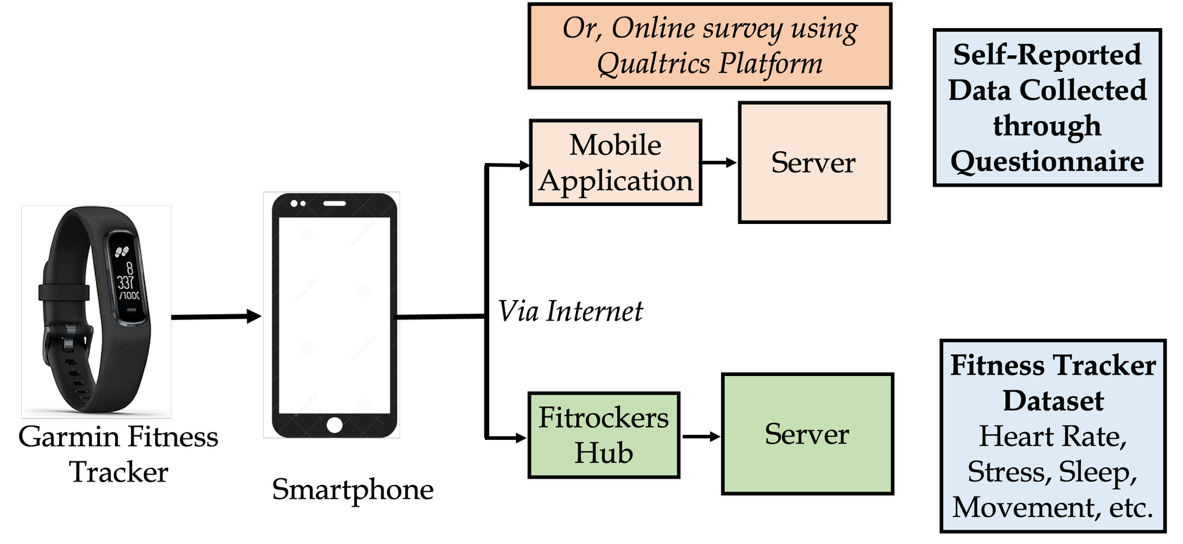


*Figure 1: A systematic block diagram of our data collection: It requires a smart watch, smartphone, FonLog mobile app or Qualtrics for self-reported data, and Fitrockr’s Hub and server for physiological data*.


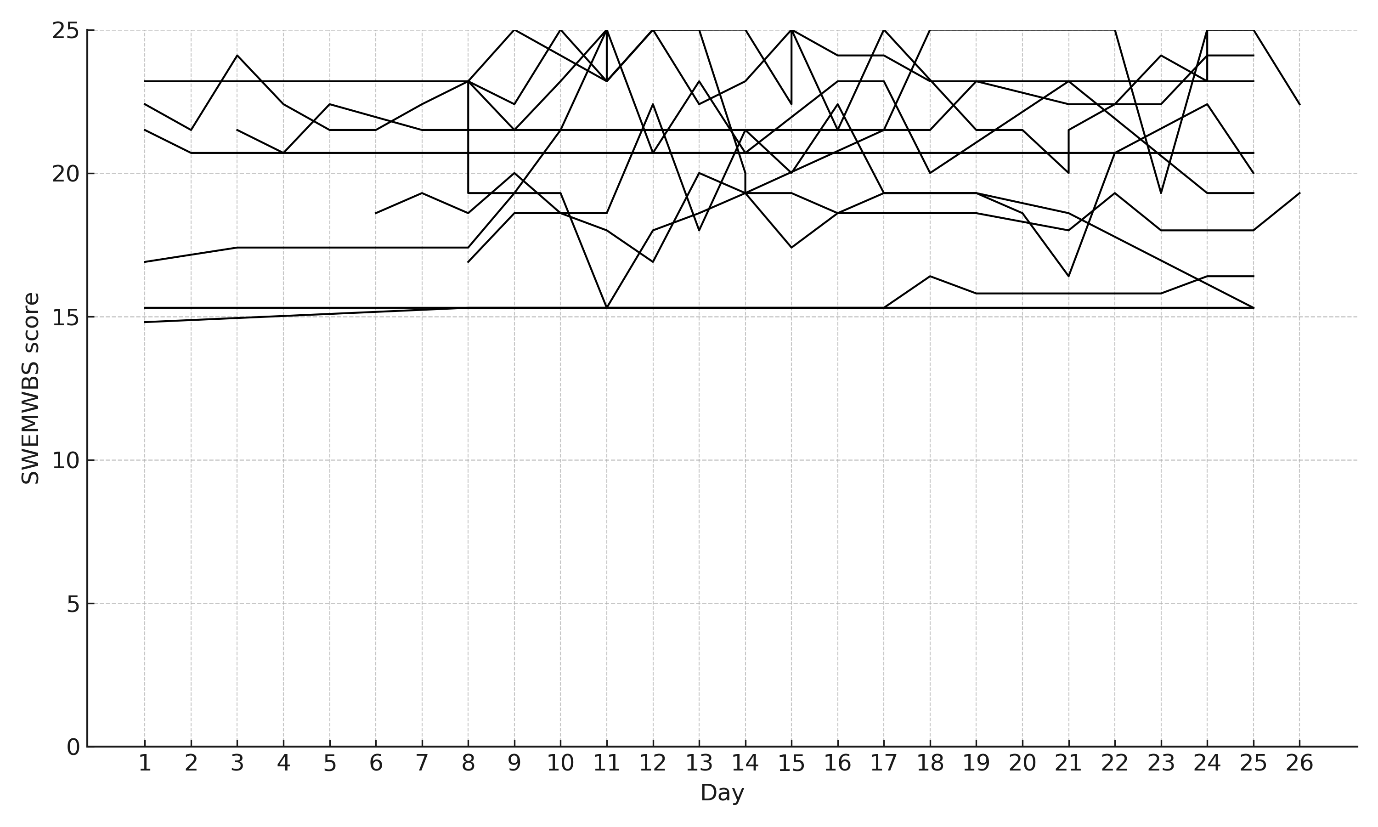


Figure 2. Observed daily SWEMWBS scores over time for each participant.


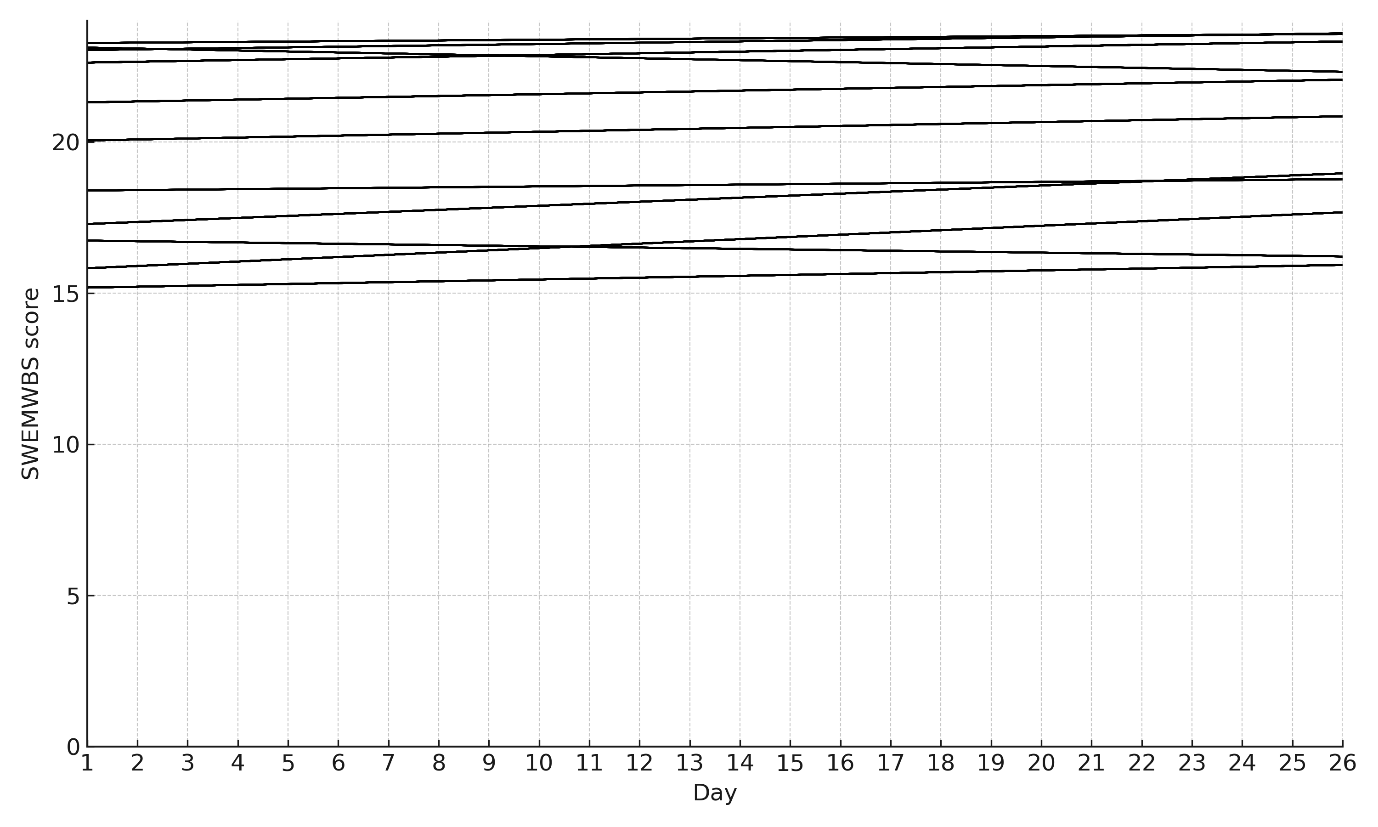


Figure 3. Predicted SWEMWBS scores over time based on individual intercept and slope estimates from the final linear mixed model.


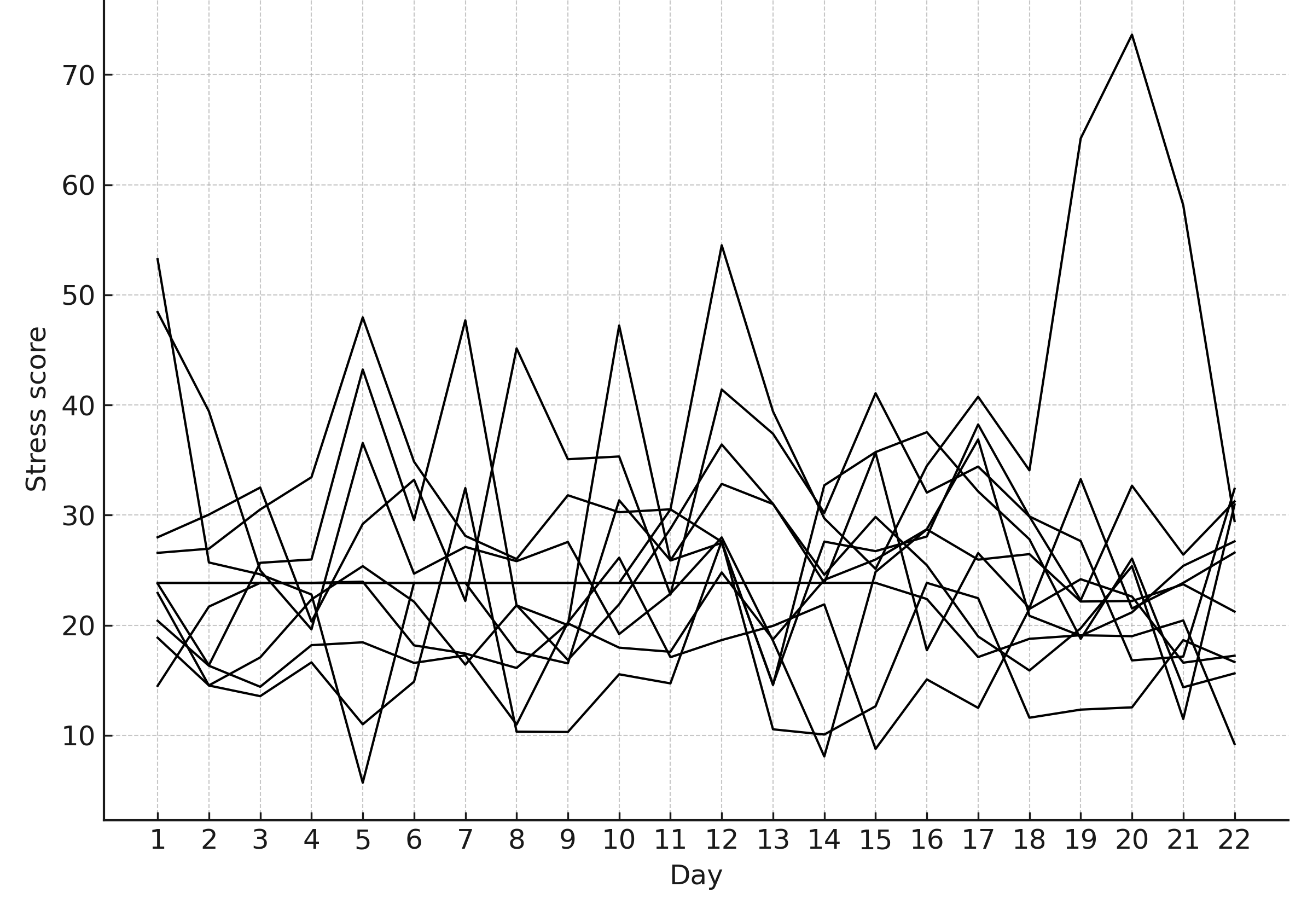


Figure 4. Observed daily stress (Heart Rate Variability) scores over time for each participant.


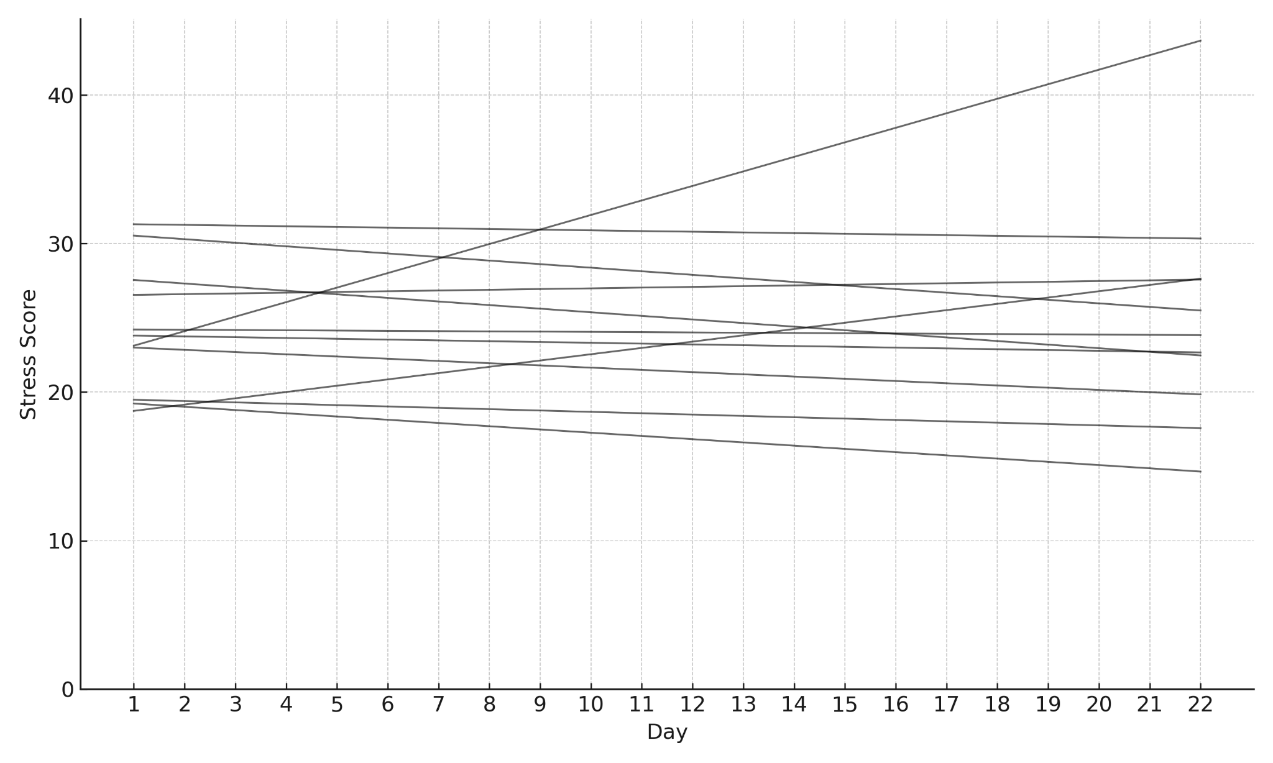


Figure 5. Predicted stress trajectories (Heart Rate Variability) based on modelled intercept and slope values for each participant.
